# Association with a novel protective microbe facilitates host adaptation to a stressful environment

**DOI:** 10.1002/evl3.223

**Published:** 2021-03-17

**Authors:** Kim L. Hoang, Nicole M. Gerardo, Levi T. Morran

**Affiliations:** ^1^ Department of Biology Emory University Atlanta Georgia 30322 USA; ^2^ Department of Zoology University of Oxford Oxford OX1 3SZ United Kingdom

**Keywords:** Experimental evolution, host adaptation, protective microbes

## Abstract

Protective symbionts can allow hosts to occupy otherwise uninhabitable niches. Despite the importance of symbionts in host evolution, we know little about how these associations arise. Encountering a microbe that can improve host fitness in a stressful environment may favor persistent interactions with that microbe, potentially facilitating a long‐term association. The bacterium *Bacillus subtilis* protects *Caenorhabditis elegans* nematodes from heat shock by increasing host fecundity compared to the nonprotective *Escherichia coli*. In this study, we ask how the protection provided by the bacterium affects the host's evolutionary trajectory. Because of the stark fitness contrast between hosts heat shocked on *B. subtilis* versus *E. coli*, we tested whether the protection conferred by the bacteria could increase the rate of host adaptation to a stressful environment. We passaged nematodes on *B. subtilis* or *E. coli*, under heat stress or standard conditions for 20 host generations of selection. When assayed under heat stress, we found that hosts exhibited the greatest fitness increase when evolved with *B. subtilis* under stress compared to when evolved with *E. coli* or under standard (nonstressful) conditions. Furthermore, despite not directly selecting for increased *B. subtilis* fitness, we found that hosts evolved to harbor more *B. subtilis* as they adapted to heat stress. Our findings demonstrate that the context under which hosts evolve is important for the evolution of beneficial associations and that protective microbes can facilitate host adaptation to stress. In turn, such host adaptation can benefit the microbe.

**Impact Summary**

Although most microbes are invisible to the naked eye, they have a profound impact on all organisms. Animals and plants that associate with microbes gain many benefits, such as nutrients or protection from enemies or harsh environments. Consequently, microbes have influenced the evolution of eukaryotic life on earth. Yet, we know very little about the evolutionary origin of these associations. Did they arise because of the host, microbe, or both? Are these initial stages driven by factors outside of the host or microbe, such as novel environmental conditions? Much of the previous work on the origin of beneficial associations relies on inferences from comparative approaches, as opposed to direct experimental tests. Unfortunately, comparative approaches do not always have the capacity to answer these questions. Here, we used a novel approach to address these challenges by evolving a host‐microbe interaction in the lab. Specifically, we evolved a eukaryotic organism (*Caenorhabditis elegans*) in the presence of a protective bacterium (*Bacillus subtilis*) under environmental (heat) stress for 20 generations. We found that the host produced the most offspring, and therefore exhibited the greatest evolutionary fitness, when evolving in the presence of the bacterium under heat stress compared to evolving in the absence of the bacterium and/or stress, and that these hosts harbored the highest bacterial abundance by the end of the experiment. Therefore, we demonstrated that a bacterium with little, if any, evolutionary history with the host facilitated the host's adaptation to a harsh environment. Further, this result suggests that traits that evolved in the host can yield benefits for *both* hosts and microbes, creating conditions that are likely to favor persistent interactions, potentially leading to long‐term association across generations (i.e., symbiosis) and even mutualism. Our work provides direct empirical evidence of factors that can drive the establishment of beneficial host‐microbe associations, thus breaking ground toward answering a fundamental but previously unaddressed set of questions.

Many eukaryotes form long‐term associations with microbes, resulting in complex symbioses that span across the tree of life. Symbionts can provide many benefits to their hosts, including production of nutrients (Douglas 1998), protection from enemies (Oliver et al. 2013), and serving as a food source (Hoang et al. 2019b). In turn, symbionts can receive benefits, such as nutrients and a hospitable environment in which to proliferate (Boettcher and Ruby 1990; Douglas 1998). These associations vary in terms of how the partnership is formed each generation, the levels of host and symbiont dependency (Fisher et al. 2017), and the level of host and symbiont exploitation (Lowe et al. 2016; Keeling and McCutcheon 2017; Sørensen et al. 2019). Indeed, symbioses are not without conflict. Theoretical models suggest that even the most ancient symbioses may have emerged from antagonistic origins (Zachar et al. 2018; Sørensen et al. 2019), and genomic evidence has shown ongoing conflict in fitness interests between host and symbiont (Sachs et al. 2011; Bennett and Moran 2015). Despite the complexities and ubiquity of symbiotic associations, most models of symbiosis originated millions of years ago. The evolutionary consequences of these partnerships are often inferred through comparison to organisms not in symbiosis (e.g., free‐living bacteria). What remains unclear is what happened at the evolutionary onset of these associations and if the factors that govern extant symbioses are representative of those that facilitate nascent associations.

The fitness outcomes of extant symbioses are often contingent on external environmental conditions (Russell and Moran 2006; Heath and Tiffin 2007; Davitt et al. 2011). Thus, external environmental conditions may also shape the evolutionary trajectory of novel interactions. Identifying the external factors or selective pressures that influence fitness outcomes of host‐microbial interactions can provide us with a better understanding of how associations arise and persist over evolutionary time (Mushegian and Ebert 2016; Gavelis and Gile 2018). External factors that facilitate long‐term association could include exposure to a new or stressful environment, which has been shown to shape the trajectories of symbioses (Klepzig et al. 2009). By themselves, hosts sometimes lack the necessary genetic variation and/or time to generate new variation, thus hindering adaptation to stressful environments. If individuals in these host populations harbored microbes that provided protection, the host populations may persist and adapt to the stress over time. Indeed, symbiont‐facilitated niche expansion is predicted to favor individual hosts that associate with protective microbes compared to hosts lacking the microbes (Kitano and Oda 2006). Further, as interactions with microbes increase host fitness through continual association, the microbes may also benefit (Lee and Ruby 1994; Bozonnet et al. 2017). Therefore, exposure to new environmental conditions may be a key factor in the establishment of novel host‐symbiont interactions, and hosts may exploit microbes as a tool to thrive in previously inaccessible environments.

Experimental evolution can serve as an important tool to understand how external factors and evolutionary processes can shape the evolutionary trajectory of interacting organisms (Hoang et al. 2016). The approach requires the use of organisms with short generation times and those that are amenable to maintenance under laboratory conditions. The nematode *Caenorhabditis elegans* is an emerging model for the study of host‐microbiome interactions (Cabreiro and Gems 2013; Berg et al. 2016; Dirksen et al. 2016; Gerbaba et al. 2017; Zhang et al. 2017), including in considering their evolutionary consequences (Ford et al. 2016, 2017; King et al. 2016; Rafaluk‐Mohr et al. 2018). Although *C. elegans* does ingest bacteria, the nematodes do not completely digest all bacteria (Portal‐Celhay and Blaser 2012; Portal‐Celhay et al. 2012): *Ochrobactrum* bacteria—a member of the natural microbiome of *C. elegans*—can remain in high abundance after hosts are removed from the bacterial source (Dirksen et al. 2016), and *Bacillus subtilis* bacteria can reside within *C. elegans* gut, resulting in benefits for the host (Gusarov et al. 2013; Donato et al. 2017). Once established, these bacteria have the potential to (co)evolve despite ingestion (Hoang et al. 2019b). Indeed, evolution experiments using *C. elegans* and bacteria have shown that increased host fitness (or at least reduced harm) can evolve in a relatively short period of time (Gibson et al. 2015; King et al. 2016). Here, leveraging the simplicity and tractability of *C. elegans*, we developed an experimental system with which to determine how the fitness of a eukaryotic host and its bacterial partner might change after multiple generations of interaction. Particularly, we dictate the conditions of the initial interaction and assess how those conditions impact the establishment of a novel association and the resulting short‐term evolutionary trajectories of host populations.

We previously found that the bacterium *B. subtilis* confers a fitness benefit to the host under heat shock compared to *Escherichia coli*, the bacterium on which *C. elegans* is maintained in the lab (Hoang et al. 2019a). Although the heat shock is a temperature that is normally very detrimental to host survival and reproduction (Aprison and Ruvinsky 2014), host reproductive fitness is greater when heat shocked on *B. subtilis*, such that hosts derive protection from the bacterium under this context (Hoang et al. 2019a). As *B. subtilis* and *C. elegans* have little prior evolutionary history (i.e., they are not known to be associated in nature or in the lab), our finding indicates that the interaction is beneficial at its initial state. Here, we ask how this protection shapes host evolution in subsequent generations. We examine how the context under which the host evolves affects its fitness when evolving with or without its microbial partner, thereby influencing the niches the host can occupy, and ultimately how these conditions shape the host's evolutionary trajectory. We experimentally evolved nematodes for 20 generations of selection under two different environmental treatments (heat stress and no heat stress), in the presence or absence of nonevolving *B. subtilis*. To determine the effects of evolving with a novel bacterium, we also evolved nematodes with a nonevolving, nonprotective *E. coli* under the two environmental conditions. After experimental evolution, we conducted fitness assays to measure host fecundity and *B. subtilis* colonization within hosts, allowing us to evaluate the effects of *B. subtilis* on host adaptation to a stressful environment. We found that hosts adapted faster in the presence of the protective bacterium under stress and that hosts spread the bacteria into the environment, setting up the potential for long‐term interactions.

## Methods

### STRAINS AND MEDIA

We independently mutated four populations of *C. elegans* N2 using ethyl methane‐sulfonate (catalog #M0880, Sigma‐Aldrich, St. Louis, MO) following Morran et al. (2011), then combined and froze the four populations to establish a single genetically variable ancestral host population, which we name LTM‐EE1. *Bacillus subtilis* strain 168 and *E. coli* strain OP50 were used as bacterial food sources. For all experiments, we grew *B. subtilis* and *E. coli* on Nematode Growth Medium Lite (US Biological, Swampscott, MA) containing 2% glucose and 0.5 mM arginine (henceforth NGMga). For steps involving GFP‐labeled OP50 (OP50‐GFP), we grew the bacterium on NGM Lite.

### EXPERIMENTAL EVOLUTION

**Figure 1:**
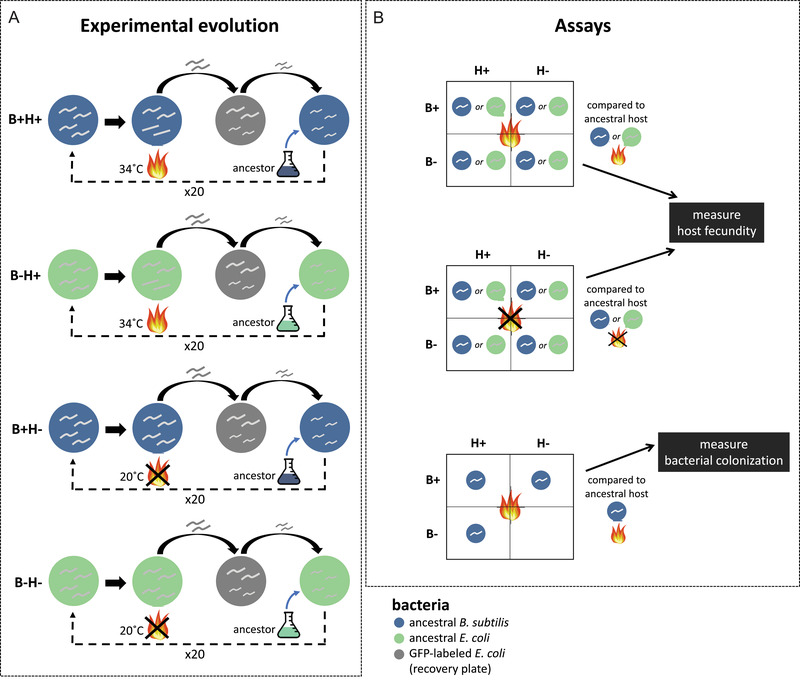
Schematic of experimental evolution and assays. (A) Nematodes were passaged on the ancestral *B. subtilis* (B+ hosts, blue) or on the ancestral *E. coli* (B– hosts, green), under heat shock (34°C, H+) or no heat shock (20°C, H–) conditions, for 20 generations of selection (40 total generations). After each heat shock, hosts recovered on GFP‐labeled *E. coli* (gray) to produce offspring. The offspring of these offspring were then placed on fresh plates of their respective bacteria to be heat shocked, starting the next generation. There were five replicate populations for each of the four treatments. (B) To measure host fecundity after 20 generations of selection, we reared hosts from each of the 20 replicate experimental populations and the ancestral population on either the ancestral *B. subtilis* or *E. coli*, then heat shocked them at 34°C or left them at 20°C, following the same schedule for one passage of experimental evolution. Two days after the heat shock, we measured the number of offspring per total number of initial adults. To measure *B. subtilis* colonization, we heat shocked 15 replicate populations (excluding the five B–H– populations) on *B. subtilis* following the schedule for one passage of experiment evolution. Immediately after heat shock, we washed and crushed nematodes and plated them on media to quantify CFUs in individual hosts.

Starting with the ancestral host population (composed of roughly 93.7% hermaphrodites and 6.3% males), we passaged the hosts under heat shock or no heat shock treatments, on either ancestral *B. subtilis* or *E. coli* (Fig. 1A). Populations in the heat shock treatment experienced heat shock every other generation for a total of 20 generations of selection (40 nematode generations total; all references to generations hereafter refer to generations of selection). A detailed protocol is available in the Supporting Information Methods. After 10 generations of selection, we froze each population, after which we thawed them again to resume the experiment. After 20 generations of selection, we again froze each replicate population, then thawed them to conduct fecundity and colonization assays. We designated populations that evolved in the presence of *B. subtilis* “B+ populations,” and those with *E. coli “*B– populations.” Likewise, populations evolved under heat stress were designated “H+ populations,” and at the standard temperature “H– populations” (Fig. 1A).

### HOST FECUNDITY

To determine host fitness changes that occurred, we quantified the number of offspring produced by nematodes after 20 generations of selection and by the ancestral host population (Fig. 1B). We followed the schedule for one passage of experimental evolution, as described in the Supporting Information Methods, for each of the evolved replicate populations (five populations from each of four experimental evolution treatments) and the ancestor, heat shocking 100–200 nematodes on either *B. subtilis* 168 or *E. coli* OP50. After heat shock, nematodes were transferred to OP50‐GFP and kept at 20°C. Two days later, we determined the number of offspring produced per heat shocked adult, which is influenced both by survival and fecundity of the surviving individuals. For each replicate population (akin to biological replicates), we heat shocked three technical replicate plates; for the ancestor, we heat shocked five replicate plates, for a total of 130 plates (4 evolved treatments × 5 replicate populations × 2 bacteria × 3 replicate plates) + (1 ancestor × 2 bacteria × 5 replicate plates). This assay was conducted for three rounds (each round included all populations from all treatments). We also quantified nematode fitness via one round of assay at generation 20 when not heat shocked, following the same procedure as the heat shock assay but keeping nematodes at 20°C throughout. To gain insight into the timing of changes in the host lineages, we similarly surveyed fecundity of the host populations after 10 generations of selection (two rounds), focusing on the two treatments where hosts evolved under heat shock with *B. subtilis* or *E. coli*.

### BACTERIAL COLONIZATION

To determine bacterial abundance within nematodes, we grew the populations evolved for 20 generations of selection (excluding the five B–H– populations because they were neither exposed to *B. subtilis* nor heat shock during their evolution) and the ancestral population on *B. subtilis* for three days, then heat shocked them for 6 h at 34°C (Fig. 1B). After heat shock, we crushed individual nematodes following a previously established protocol to determine *B. subtilis* abundance (Vega and Gore 2017). A detailed protocol is found in the Supporting Information Methods. We heat shocked one plate for each replicate population, and three replicate plates for the ancestor. From each population, we crushed 10 individuals separately to quantify abundance of *B. subtilis* within a single nematode and the frequency at which each host was colonized. In total, we quantified bacterial abundance for 180 individuals (3 evolved treatments × 5 replicate populations × 10 individuals) + (1 ancestor × 3 populations × 10 individuals). We conducted this assay for four rounds.

### STATISTICAL ANALYSES

We analyzed the total fecundity means (i.e., the mean of each replicate population from each experimental evolution treatment, determined by averaging the technical replicates for a given population within a given round) for populations heat shocked after generation 20 using a linear mixed model. The main effects of experimental evolution bacteria (*B. subtilis or E. coli*), experimental evolution environment (heat shock or no shock), and their interaction were treated as fixed terms. Round and replicate population (nested within experimental evolution bacteria and environment) were treated as random effects. We then performed Student's *t*‐tests (pairwise post hoc tests within the analysis) to compare means between treatments. We conducted separate analyses for each assay substrate (*B. subtilis* and *E. coli*), as running separate models substantially improved the model fit as determined by AICc (including the assay substrate within our model did not qualitatively alter the results nonetheless). We also performed linear mixed model analyses for populations at generation 10 and populations that were assayed under standard conditions (no heat shock) after generation 20. These models included testing experimental evolution bacteria, experimental evolution environment, and their interaction as fixed terms, with replicate population (nested within experimental evolution bacteria and environment) as a random term. Finally, round was also included as a random term in the generation 10 model, but not included in the model for the generation 20 populations that were assayed under no shock because only one round of data was collected for that assay.

To analyze the colony‐forming unit (CFU) abundance per host data, we used a linear mixed model to assess the total CFU means, determined by averaging across 10 nematodes from each replicate population within a given round. We treated the main effect of evolutionary treatment (B+H+, B–H+, and B+H–) as a fixed effect. Round, replicate population (nested within experimental evolution bacteria and environment), and evolutionary treatment × round were treated as random effects. We then performed Student's *t*‐tests to compare means between treatments. For the frequency of colonized hosts data, we converted the CFU counts to binomial data (presence/absence of CFUs) and used a Fisher's exact test. For pairwise comparisons, we conducted a separate generalized linear model (GLM), with a binomial distribution and logit link function implemented using Firth adjusted maximum likelihood. We tested evolutionary treatment (as above) and replicate population (nested within evolutionary treatment) as fixed effects. We then performed linear contrast tests (mechanisms within the analysis for comparing variables within a fixed effect in the mixed model) to compare between treatments. We did not include the ancestral hosts directly in our analyses because the ancestral population was a single population without replicates and could not be analyzed while testing the effect of replicate population. Finally, to determine the association between host fecundity and *B. subtilis* colonization, we fit the average fecundity by average CFUs for each replication population across B+H+, B–H+, and B+H– hosts heat shocked on *B. subtilis* with a linear function. All analyses were conducted using JMP Pro 14.

### BACTERIAL SPORE FORMATION AND SPREAD IN NEW ENVIRONMENT

Because spores cannot be digested by nematodes (Donato et al. 2017), we asked whether *B. subtilis* forms spores in evolved hosts. We picked adult nematodes from the ancestral population and one B+H+ population (the one with the greatest fecundity and CFU count on *B. subtilis*) that were heat shocked or not heat shocked into M9, then crushed and incubated them at 80°C for 20 min, which would kill all vegetative cells but not spores (Gusarov et al. 2013). We then plated the mixture onto LB and quantified the number of CFUs (2 populations × 2 assay environments × 3 replicate plates × 10 individuals per plate).

To determine whether evolved nematodes can spread *B. subtilis* in new environments, we picked heat shocked B+H+ hosts onto unseeded NGMga plates and monitored bacterial growth and nematode population size on the plate for a week at the standard rearing temperature (five replicate plates, 10 individuals per plate).

## Results

### HOST FECUNDITY

We determined whether hosts evolved under different conditions exhibited differences in fecundity. By assaying hosts in the conditions under which they evolved, in addition to conditions that other host treatments experienced, we were able to distinguish host adaptation to heat stress versus the evolution of greater protection derived from *B. subtilis* association.

**Figure 2:**
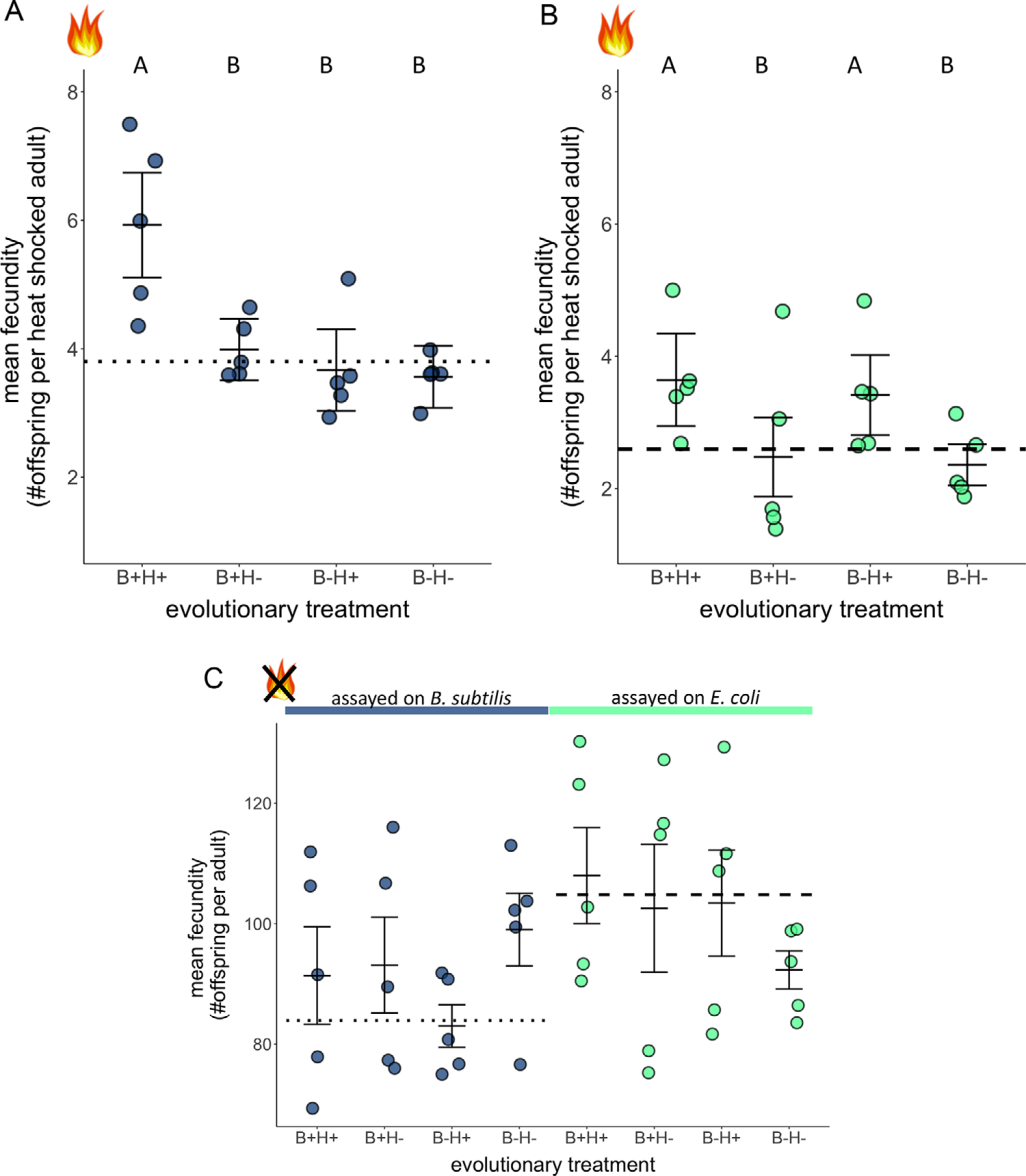
Fecundity of evolved hosts after 20 generations of selection. The *x*‐axis indicates the condition under which nematodes evolved. Nematodes from the four experimental treatments were heat shocked at 34°C on (A) *B. subtilis* or (B) *E. coli*. Each plate contained roughly 200 nematodes. The data are combined across three rounds. (C) Nematodes from the four experimental treatments were kept at 20°C on *B. subtilis* (blue points) or *E. coli* (green points). Each plate contained roughly 150 nematodes. The dotted line and dashed line indicate the average value for the ancestral host on *B. subtilis* and *E. coli*, respectively. Error bars indicate the standard errors. Treatments that are not the same letter are significantly different. Note the *y*‐axes for panels A and B versus panel C differ by almost 20‐fold. Figure S2 shows the distribution of all individual data points for this figure.

When we surveyed host fitness after 10 generations, hosts that evolved with or without *B. subtilis* did not differ in terms of fecundity when heat shocked on *B. subtilis* (*F*_(1,13)_ = 3.77, *P* = 0.074; Fig. S1) or on *E. coli* (*F*_(1,13)_ = 0.0007, *P* = 0.98; Table S1). After 20 generations, however, we found that there was a significant interaction between experimental evolution bacteria and environment (*F*_(1,16)_ = 5.98, *P* = 0.03; Table S2). Across hosts that were assayed on *B. subtilis*, nematodes that evolved with *B. subtilis* under heat stress (B+H+) exhibited greater fecundity compared to B+H– hosts (Fig. 2A; first vs. second treatment; Student's *t*‐test = 3.66, *P* = 0.002; Table S3), indicating that evolution with the protective bacterium was not sufficient for host adaptation; rather, the stressful environment plays a key role in the increased fecundity of B+H+ hosts. Similarly, B–H+ hosts did not adapt as well as B+H+ hosts (Fig. 2A; first vs. third treatment; Student's *t*‐test = 4.27, *P* = 0.0006), demonstrating that solely evolving under heat stress was not sufficient—evolution with the protective microbe is also necessary.

To determine the extent to which the increased fecundity of these hosts was driven by the nematodes themselves, we also heat shocked all hosts on *E. coli*. Here, we found that there was an effect of the experimental evolution environment, where B+H+ and B–H+ hosts exhibited greater fecundity than B+H– and B–H– hosts (Fig. 2B; first and third treatment vs. second and fourth treatment, *F*_(1,16)_ = 6.64, *P* = 0.02; Table S2). This finding indicates that hosts that evolved under heat stress adapted to the novel environment. Importantly, we did not detect significant effects of evolution with either *B. subtilis* or *E. coli*, or an interaction between the bacteria and the environment (*F*_(1,16)_ = 0.16, *P* = 0.69; *F*_(1,16)_ = 0.016, *P* = 0.90, respectively). Therefore, B+H+ hosts were not different from B–H+ hosts in the absence of *B. subtilis*, demonstrating that exposure to *B. subtilis* was critical for the elevated fecundity exhibited by the B+H+ populations (Fig. 2A). These results show that exposure to both the beneficial bacterium (during evolution and during heat shock) and the stressful environment together was necessary to facilitate increased levels of host adaptation to the heat shock.

In parallel assays without heat shock after generation 20, we found that exposure to *B. subtilis* versus *E. coli* significantly altered host reproduction (Fig. 2C; *F*_(1,19)_ = 4.87, *P* = 0.04; Table S4). Hosts produced greater quantities of offspring on *E. coli* compared to *B. subtilis*. However, there were no significant effects of experimental evolution conditions on host fecundity (bacterial exposure during evolution, *F*_(1,16)_ = 0.49, *P* = 0.49; environment during evolution, *F*_(1,16)_ = 0.002, *P* = 0.96; interaction between bacteria and environment, *F*_(1,16)_ = 0.12, *P* = 0.73). Despite nematodes evolving the greatest overall fecundity on *B. subtilis* under heat stress, they did not gain a similar proportional increase in fitness when the heat shock was removed at generation 20. Thus, the benefits of evolving in the presence of *B. subtilis* were contextual and limited to the heat shock environment.

### BACTERIAL COLONIZATION

**Figure 3:**
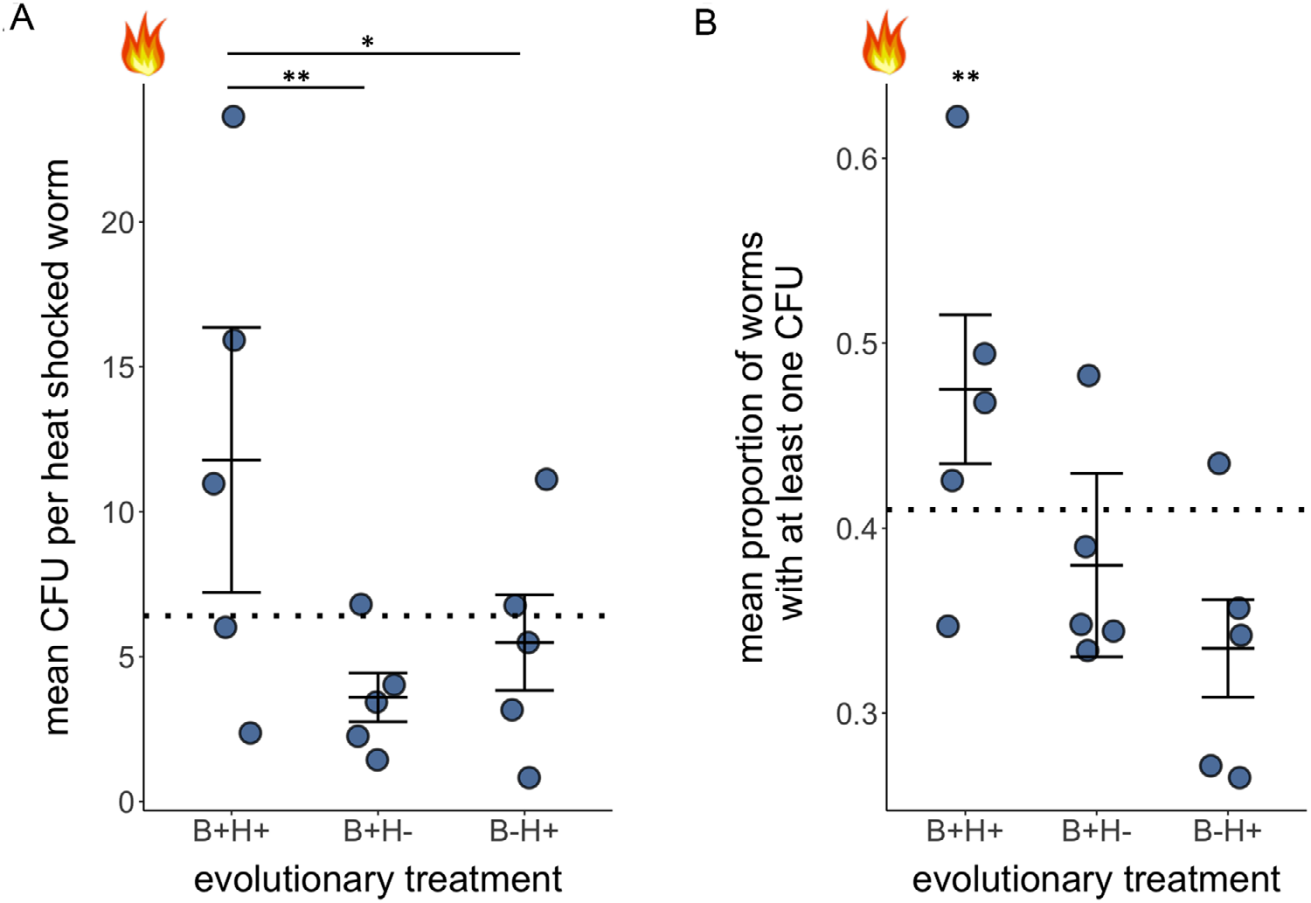
*Bacillus subtilis* colonization in evolved hosts. Evolved nematodes were heat shocked on *B. subtilis*, washed, and individually crushed to quantify within‐host bacterial colonization. The *x*‐axis indicates the condition under which nematodes evolved. (A) The number of colony‐forming units (CFUs) in each nematode. (B) The proportion of nematodes harboring at least one CFU. Each data point is the average of 10 nematodes from each replicate population from experimental evolution. The data are combined across four rounds. The dotted line indicates the average value for the ancestral host. Error bars indicate the standard errors. ^**^ *P*< 0.01^, *^ *P* < 0.05.

**Figure 4:**
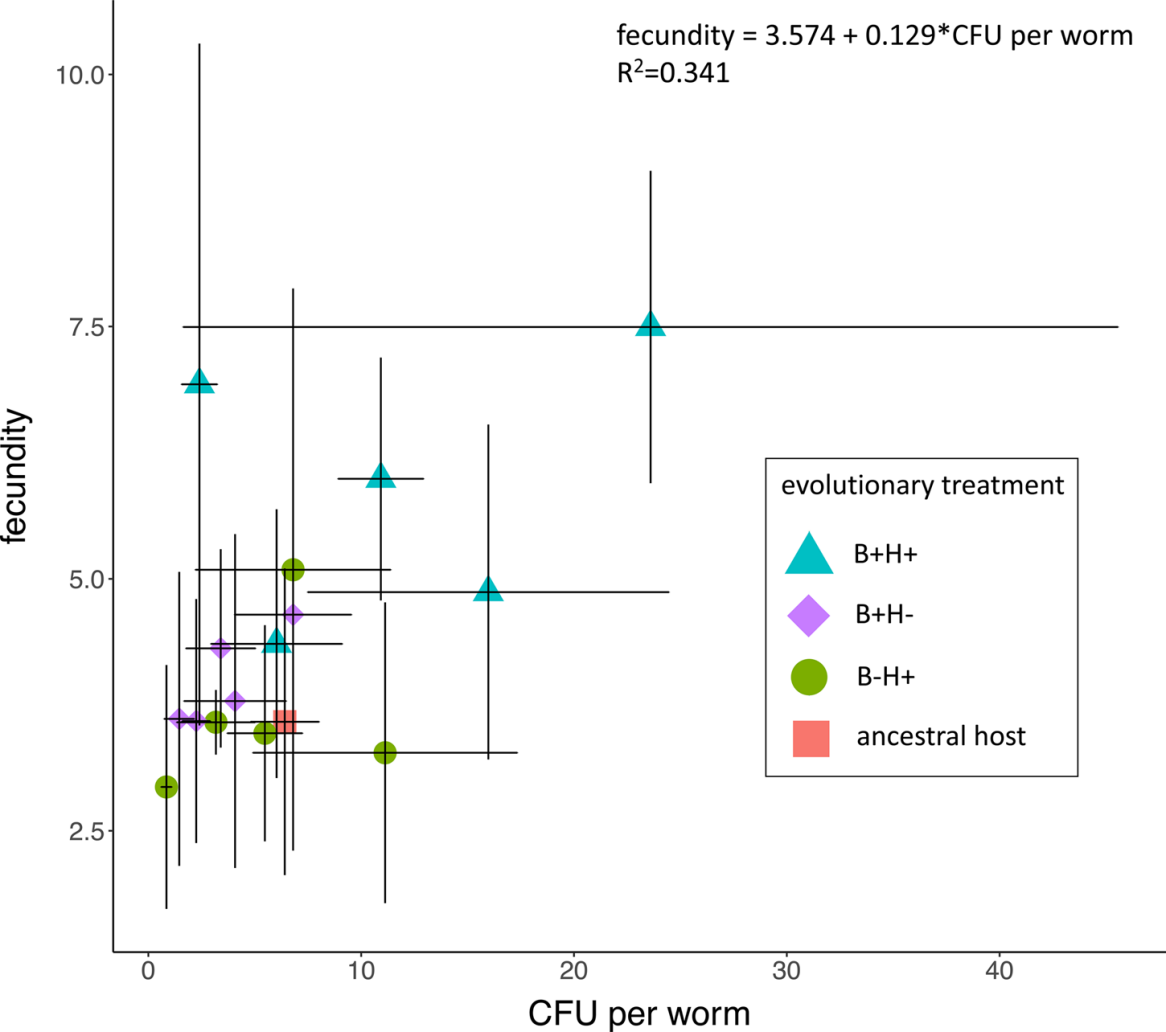
Host fecundity versus *B. subtilis* colonization when heat shocked after generation 20. Fecundity is plotted against CFUs per host. Error bars indicate standard errors.

To determine whether differences in host fitness were associated with changes in *B. subtilis* colonization, we quantified *B. subtilis* abundance and the frequency of colonization in the evolved hosts when heat shocked after generation 20. We found significant treatment differences in the number of CFUs (*F*_(3,66)_ = 6.57, *P* < 0.001) and in the proportion of nematodes harboring *B. subtilis* (Fig. 3; $\chi_{2}^{2}$ = 9.45, *P* = 0.009; Fisher's exact test *P* = 0.015; Tables S5–S7). Specifically, B+H+ hosts harbored more CFUs than B+H– (Student's *t*‐test = 3.18, *P* = 0.002) and B–H+ (Student's *t*‐test = 2.44, *P* = 0.02) hosts. B+H+ hosts also had a greater proportion of nematodes harboring the bacterium (Fig. 3B; $\chi_{1}^{2}\;$= 9.48, *P* = 0.002). We then plotted host fecundity against *B. subtilis* CFUs (Fig. 4). Overall, we found that increased *B. subtilis* abundance was positively correlated with increased host reproduction (*R* ^2^ = 0.34; *F*_1,14_ = 7.23, *P* = 0.018; Table S8), where B+H+ hosts had the highest fecundity and *B. subtilis* abundance compared to the ancestor and other evolved hosts when heat shocked after generation 20.

### BACTERIAL SPORE FORMATION AND SPREAD IN NEW ENVIRONMENT

Under experimental conditions in which only spore‐forming bacteria would be able to persist, no bacterial growth was observed when nematodes were heat shocked, indicating that *B. subtilis* does not form spores in stressed hosts (Table S9). However, we found that heat shocked hosts were able to disperse *B. subtilis* in a new environment (unseeded NGMga plates), where novel bacterial growth expanded out from the spot where nematodes were introduced. Moreover, after approximately two host generations, three out of five of these replicate plates contained several hundred larvae (Fig. S3).

## Discussion

Eukaryotes have gained numerous advantages from long‐term association with their symbionts, associations that have been shaped by a series of intricate processes since their establishment. For example, one of the most important symbioses in eukaryotic evolution, the mitochondrion, is hypothesized to have evolved from a bacterium that was phagocytized by an archaeon, where the bacterium remained as farmed prey until it became a permanent endosymbiont (Maynard Smith, J. and Szathmary 1995; Zachar et al. 2018). For many other ancient symbioses, it has been difficult to elucidate their origin because of the profound changes that have occurred over the course of their evolution.

Work on extant symbioses has shown that context dependency plays a large part in the maintenance and exchange of benefits between hosts and symbionts (Heath and Tiffin 2007; Weldon et al. 2013; Keeling and McCutcheon 2017). Facilitation of host adaptation to a new or stressful environment by a microbe may be a way in which novel associations begin, where the symbiont provides its host with abilities it previously lacked (Douglas 2014). By taking advantage of a novel microbe, the host can expand its genetics toolbox. In this study, we examined how evolution in the presence of a protective bacterium affected host adaptation to a stressful environment. In the absence of environmental stress, evolved nematodes produced fewer offspring on *B. subtilis* compared to *E. coli* (Fig. 2C). Importantly, we found that nematodes evolved in the presence of a protective bacterium (B+H+ hosts) exhibited the greatest increase in post‐heat shock fecundity after generation 20 when exposed to the protective microbe (Fig. 2A, first treatment). Because hosts did not show signs of improved fitness at generation 10 (Fig. S1), increased fecundity at generation 20 provides evidence for host evolution instead of transgenerational effects, as would be suggested from past studies on *C. elegans* subjected to high temperatures (Klosin et al. 2017). This nonlinear increase in fitness over the course of the experiment is not surprising given that beneficial alleles were likely at low initial frequencies (Muller 1932; Lenski et al. 1991).

Our findings indicate that presence of both the protective bacterium and the stressful environment were critical for facilitating host adaptation (Fig. 2A). Additionally, adaptation was not solely driven by an overall increase in heat tolerance on the part of the host, but rather by the host's usage of the protective bacteria (Figs. 2A and 2B). A potential molecular mechanism may involve modulation of the host's stress response by *B. subtilis*. Past research has found that heat shock response in the host resulted in increased survival but decreased reproductive fitness (Casanueva et al. 2012), whereas a separate study found that *B. subtilis* decreased the host's heat shock response with a moderate increase in temperature (Gómez‐Orte et al. 2018). Taken together, B+H+ hosts may have evolved reduced expression of host stress response genes in the presence of *B. subtilis*, resulting in increased fecundity after heat shock. Ultimately, although we do not know the exact mechanism, the presence of a protective bacteria in a stressful environment altered the evolutionary trajectories of host populations, facilitating host adaptation.

Unexpectedly, the fitness benefits of microbial‐facilitated host adaptation were not restricted to only the host populations. Although we did not select for increased *B. subtilis* colonization throughout our experiment, we found that B+H+ hosts allowed for increased within‐host *B. subtilis* growth and a greater propensity to be colonized by *B. subtilis* (Fig. 3). Furthermore, these bacteria persisted in the presence of nematodes and spread into the environment (Fig. S3), a feature of extant symbioses in which bacteria are ingested (Kikuchi et al. 2007; Brock et al. 2011). Therefore, both the host and microbe benefited from host adaptation, despite the fact that *B. subtilis* did not evolve during the experiment. This demonstrates that a host's microbial partner can directly benefit from host evolution, even without evolving itself. Although changes to host or microbial fitness may often be influenced heavily by microbial evolution (Ford et al. 2016; King et al. 2016), our study presents a case in which changes in the host affected both its own fitness and that of its microbe. These data indicate that initial host evolution may play a critical role in the establishment of novel beneficial associations between hosts and microbes. There could be several mechanisms involved in the greater number of CFUs in evolved hosts: nematodes could eat more, allow more live bacteria to pass through the nematode grinder, or suppress microbial regulation in the gut. Regardless of the underlying mechanism, we found elevated levels of live *B. subtilis* inside hosts after heat shock (Fig. 3). These bacteria have the potential to colonize and spread into the environment. This greater propensity for hosts to harbor live microbes could have substantial long‐term benefits for bacteria. If host adaptation permits greater bacterial colonization, essentially creating a novel niche for the microbe, then selection could favor host‐associated microbes and result in greater microbial fitness. However, colonization is not necessary for the bacteria to derive host‐associated benefits. Indeed, hosts are typically more mobile than microbes, and association with the host could facilitate microbial dispersal when microbes exit their host and proliferate in the external environment (Lee and Ruby 1994; Brock et al. 2011; Thutupalli et al. 2017).

Despite observing conditional adaptation of the B+H+ hosts and increased microbial fitness within B+H+ hosts, we found substantial variation in host fecundity and bacterial colonization both across and within host populations (Figs. 2, 3, and S2). This variation may be a product of the host, the protective microbe, or their interaction. Variance due to the host may have resulted from substantial levels of standing genetic variation in our ancestral host population that may have been maintained over the course of the experiment. However, microbial establishment likely also plays a role in the manifestation of fecundity and CFU variance. Variation in *B. subtilis* colonization is consistent with previous research in within‐host bacterial growth in *C. elegans*, where stochasticity is an important factor in determining microbial community composition between individual hosts, even when those hosts are genetically identical (Vega and Gore 2017). As a whole, we observed substantial variation in host populations that exhibited the greatest increase in reproduction and *B. subtilis* colonization (i.e., B+H+ hosts). Portions of these populations have evolved the ability to maximize their fitness under heat stress with the aid of *B. subtilis*, but these traits have seemingly not fixed in the population. Conversely, traits permitting hosts to derive greater protection from *B. subtilis* colonization may have swept through the host population, but stochasticity in bacterial colonization generated variance in fecundity. Nonetheless, the amount of variation present may diminish if these populations were to continue evolving as favorable host traits sweep to fixation or hosts evolve even greater propensity for *B. subtilis* colonization.

We acknowledge that, in our system, it may be difficult to disentangle the roles of the bacteria as a food or as a nonfood partner. It is in large part because of the ease with which *C. elegans* interacts with bacteria through diet that we used in this system to study the evolutionary consequences of beneficial interactions—the nematodes have no choice but to physically interact with the bacteria. Ingestion in itself could be the benefit the host receives, but that does not disqualify this interaction as potentially symbiotic. At the very least, more bacteria in passaged nematodes indicate that hosts have evolved to harbor more of their food source, but a food source that they do not immediately digest, indicating that there is potential for further interactions to evolve. There are several lines of evidence supporting the role of *B. subtilis* as a model for the study of incipient symbiosis. First, symbionts need not be distinguished from food. In many extant symbioses, symbionts serve primarily as the host's food source (e.g., fungus‐farming insects [Weber 1966; Aanen et al. 2002; Six 2012; Menezes et al. 2015]; bacteria‐farming amoebae [Brock et al. 2011]). Second, in other symbioses, symbionts that are food also serve other important roles (Kukor and Martin 1983; Forst et al. 1997; Lindquist et al. 2005; Kodama and Fujishima 2008; Jäckle et al. 2019). Third, although the majority of consumed *B. subtilis* are digested by the host, live *B. subtilis* colonizes the host gut after consumption (Donato et al. 2017; Hoang et al. 2019a). The importance of live bacteria in host development is also supported by previous research, which has shown that eggs hatched on heat‐killed bacteria are arrested at an early larval stage (Ruaud and Bessereau 2006). Fourth, a long‐term association can evolve despite a high turnover of bacteria within the host. For example, in the bobtail squid‐*Vibrio fischeri* association, the host squid expels 95% of its bacteria each day, yet this is still considered a canonical, protective symbiosis that has shaped host evolution (Ruby 1996; McFall‐Ngai 2014). Although the turnover in the squid‐*Vibrio* system is due to expulsion instead of consumption, the persistence of the association relying on maintenance of only a small subset of the microbial population is quite similar. Therefore, like known established symbionts, *B. subtilis* has the potential to form a beneficial association with its host, even though it is also a food source. Moreover, despite the low number of individual hosts (10) that dispersed to a new environment (unseeded NGMga plate), this was enough for bacteria to spread and support growth of subsequent host generations (Fig. S3).

Our study sheds light into the conditions under which novel beneficial associations may arise. Evolution with a protective bacterium could either enhance or hinder host adaptation (Bennett and Moran 2015; Martinez et al. 2016). Martinez et al. 2016 demonstrated that evolution of *Drosophila* resistance to a viral pathogen was impeded by association with a protective *Wolbachia* bacterium (Martinez et al. 2016). In our study, we examined how a host‐microbe interaction evolves when the association is initially protective for the host under stress. We found that associating with the bacterium led to hosts gaining a fitness advantage under heat stress, which allowed them to occupy a hostile environment better than their counterparts that evolved without the protective partner. Our results contrasted those of Martinez et al. (2016) in that evolution with the protective bacterium did not impede host adaptation. Indeed, we demonstrate that a bacterium with little, if any, evolutionary history with the host facilitated its adaptation. Specifically, although hosts that evolved with *B. subtilis* under stress exhibited the greatest fitness, they did not incur a drastic decrease in fitness when heat shocked in the absence of the protective microbe after generation 20 (Fig. 2B). These findings, in turn, provide directions for future exploration of the system—how the association can be maintained over evolutionary time. Are hosts evolving to harbor and propagate more of a nutritional resource that can make them more robust to environmental challenges, a protective microbe that induces physiological changes in the face of stressors, or both? Eventually, increased fitness from associating with the microbe may lead to increased dependency, further reinforcing interactions between host and microbe. Increased microbial fitness within hosts can evolve over time because the host can provide its symbiont with a more optimal environment than the external environment, such as nutrient availability and fewer competitors, even when the microbe is initially exploited in the evolution of the association (Davidson et al. 2004; Wilson et al. 2010; Bozonnet et al. 2017; Sørensen et al. 2019). Once the partners evolve to depend on one another, hosts and microbes that associate with one another gain a fitness advantage over those that lack a partner. These short‐term evolutionary effects may then promote reciprocal adaptation and coevolution. Ultimately, use and optimization of novel microbes by hosts may provide important stepping stones toward the evolution of obligate dependency and long‐term beneficial symbioses.

## DATA ARCHIVING

The data that support the findings of this study are openly available in Dryad Data Repository at https://doi.org/10.5061/dryad.dv41ns1xg

## AUTHOR CONTRIBUTIONS

KLH, NMG, and LTM conceived the study and designed the experiments. KLH performed the experiments. KLH and LTM analyzed the data. KLH wrote the manuscript with input from NMG and LTM.

## CONFLICT OF INTEREST

The authors declare no conflict of interest.

Associate Editor: C. Moreau

## Supporting information


**Table S1**. Fixed effects and random effects summary for linear mixed models of host fecundity when heat shocked on either *B. subtilis* or *E. coli* after 10 generations.
**Table S2**. Fixed effects and random effects summary for linear mixed models of host fecundity when heat shocked on either *B. subtilis* or *E. coli* after 20 generations.
**Table S3**. Summary of pairwise comparisons between treatments for fecundity of hosts heat shocked on *B. subtilis* after 20 generations.
**Table S4**. Fixed effects and random effects summary for linear mixed model of host fecundity assayed without heat shock on either *B. subtilis* or *E. coli* after 20 generations.
**Table S5**. Fixed effects and random effects summary for linear mixed model of *B. subtilis* abundance.
**Table S6**. Whole table summary for GLM of frequency of hosts harboring *B. subtilis*.
**Table S7**. Effect tests for GLM of frequency of hosts harboring *B. subtilis*.
**Table S8**. ANOVA table for host fecundity vs. *B. subtilis* CFU.
**Table S9**. *B. subtilis* spore formation in heat shocked and non‐heat shocked nematodes.
**Figure S1**. **Fecundity means of evolved hosts after ten generations of selection**
**Figure S2**. **Distribution of data points for fecundity means of evolved hosts in Figure 2** Each data point is the average of three replicate plates for each of the five replicate populations from experimental evolution. The x‐axis indicates the condition under which nematodes evolved.
**Figure S3**. **Spread of *B. subtilis* by hosts into new environment** After heat shock, 10 B+H+ hosts were transferred by platinum wire onto an unseeded NGM plate.
