# Supplementary material for: Association with a novel protective microbe facilitates host adaptation to a stressful environment
Source: Evol Lett. 2021 Mar 17;5(2):118–29. doi: 10.1002/evl3.223 (PMC8045907; doi:10.1002/evl3.223)
Supplement: Supplementary file 1 — Table S1. Fixed effects and random effects summary for linear mixed models of host fecundity when heat shocked on either B. subtilis or E. coli after 10 generations. Table S2. Fixed effects and random effects summary for linear mixed models of host fecundity when heat shocked on either B. subtilis or E. coli after 20 generations. Table S3. Summary of pairwise comparisons between treatments for fecundity of hosts heat shocked on B. subtilis after 20 generations. Table S4. Fixed effects and random effects summary for linear mixed model of host fecundity assayed without heat shock on either B. subtilis or E. coli after 20 generations. Table S5. Fixed effects and random effects summary for linear mixed model of B. subtilis abundance. Table S6. Whole table summary for GLM of frequency of hosts harboring B. subtilis. Table S7. Effect tests for GLM of frequency of hosts harboring B. subtilis. Table S8. ANOVA table for host fecundity vs. B. subtilis CFU. Table S9. B. subtilis spore formation in heat shocked and non‐heat shocked nematodes. Figure S1. Fecundity means of evolved hosts after ten generations of selection Figure S2. Distribution of data points for fecundity means of evolved hosts in Figure 2 Each data point is the average of three replicate plates for each of the five replicate populations from experimental evolution. The x‐axis indicates the condition under which nematodes evolved. Figure S3. Spread of B. subtilis by hosts into new environment After heat shock, 10 B+H+ hosts were transferred by platinum wire onto an unseeded NGM plate. [file EVL3-5-118-s001.docx]

**Supplemental Information**

**Table S1.** Fixed effects and random effects summary for linear mixed models of host fecundity when heat shocked on either *B. subtilis* or *E. coli* after 10 generations*.*

| **Assay** |  | **Source** | **#**  **parameters** | **DF**  **numerator** | **DF**  **denominator** | **F Ratio** | **Prob > F** |  | **Variance Component** | **Estimate** | **Std Error** | **Wald p-Value** |
| --- | --- | --- | --- | --- | --- | --- | --- | --- | --- | --- | --- | --- |
| *B. subtilis* | **Fixed effects** | evolution bacteria | 1 | 1 | 13.0 | 3.77 | 0.07 | **Random effects** | round | -0.03 | 0.02 | 0.06 |
|  |  |  |  |  |  |  |  |  | Residual | 0.31 | 0.12 |  |
|  |  |  |  |  |  |  |  |  | Total | 0.31 | 0.12 |  |
| *E. coli* | **Fixed effects** | evolution bacteria | 1 | 1 | 13.0 | 0.0007 | 0.98 | **Random effects** | round | 0.07 | 0.11 | 0.52 |
|  |  |  |  |  |  |  |  |  | Residual | 0.06 | 0.02 |  |
|  |  |  |  |  |  |  |  |  | Total | 0.13 | 0.11 |  |

**Table S2**. Fixed effects and random effects summary for linear mixed models of host fecundity when heat shocked on either *B. subtilis* or *E. coli* after 20 generations*.*

| **Assay** |  | **Source** | **#**  **parameters** | **DF**  **numerator** | **DF**  **denominator** | **F Ratio** | **Prob > F** |  | **Variance Component** | **Estimate** | **Std Error** | **Wald p-Value** |
| --- | --- | --- | --- | --- | --- | --- | --- | --- | --- | --- | --- | --- |
| *B. subtilis* | **Fixed effects** | evolution environment | 1 | 1 | 16.0 | 7.48 | 0.01* | **Random effects** | population[evolution environment, evolution bacteria] | -0.25 | 0.33 | 0.45 |
|  |  | evolution bacteria | 1 | 1 | 16.0 | 12.88 | 0.003* |  | round | 4.34 | 4.49 | 0.33 |
|  |  | evolution environment * evolution bacteria | 1 | 1 | 16.0 | 5.98 | 0.03* |  | Residual | 2.85 | 0.65 |  |
|  |  |  |  |  |  |  |  |  | Total | 7.19 | 4.53 |  |
| *E. coli* | **Fixed effects** | evolution environment | 1 | 1 | 16.0 | 6.64 | 0.02* | **Random effects** | population[evolution environment, evolution bacteria] | -0.03 | 0.40 | 0.94 |
|  |  | evolution bacteria | 1 | 1 | 16.0 | 0.16 | 0.69 |  | round | 2.87 | 3.01 | 0.34 |
|  |  | evolution environment * evolution bacteria | 1 | 1 | 16.0 | 0.02 | 0.90 |  | Residual | 2.88 | 0.66 |  |
|  |  |  |  |  |  |  |  |  | Total | 5.75 | 3.08 |  |

**Table S3**. Summary of pairwise comparisons between treatments for fecundity of hosts heat shocked on *B. subtilis* after 20 generations.

| evolution environment | evolution bacteria | - evolution environment | - evolution bacteria | t Ratio | Prob>\|t\| |
| --- | --- | --- | --- | --- | --- |
| heat shock | *B. subtilis* | heat shock | *E. coli* | 4.27 | 0.0006* |
| heat shock | *B. subtilis* | no heat shock | *B. subtilis* | 3.66 | 0.002* |
| heat shock | *B. subtilis* | no heat shock | *E. coli* | 4.47 | 0.0004* |
| heat shock | *E. coli* | no heat shock | *B. subtilis* | -0.60 | 0.55 |
| heat shock | *E. coli* | no heat shock | *E. coli* | 0.20 | 0.84 |
| no heat shock | *B. subtilis* | no heat shock | *E. coli* | 0.81 | 0.43 |

**Table S4**. Fixed effects and random effects summary for linear mixed model of host fecundity assayed without heat shock on either *B. subtilis* or *E. coli* after 20 generations*.*

|  | **Source** | **#**  **parameters** | **DF**  **numerator** | **DF**  **denominator** | **F Ratio** | **Prob > F** |  | **Variance Component** | **Estimate** | **Std Error** | **Wald p-Value** |
| --- | --- | --- | --- | --- | --- | --- | --- | --- | --- | --- | --- |
| **Fixed effects** | evolution environment | 1 | 1 | 16.0 | 0.002 | 0.96 | **Random effects** | population[evolution environment, evolution bacteria] | 88.17 | 74.66 | 0.24 |
|  | evolution bacteria | 1 | 1 | 16.0 | 0.49 | 0.49 |  | Residual | 202.79 | 65.79 |  |
|  | evolution environment * evolution bacteria | 1 | 1 | 16.0 | 0.12 | 0.73 |  | Total | 290.95 | 74.66 |  |
|  | assay bacteria | 1 | 1 | 19.0 | 4.87 | 0.04* |  |  |  |  |  |

**Table S5**. Fixed effects and random effects summary for linear mixed model of *B. subtilis* abundance*.*

|  | **Source** | **#**  **parameters** | **DF**  **numerator** | **DF**  **denominator** | **F Ratio** | **Prob > F** |  | **Variance Component** | **Estimate** | **Std Error** | **Wald p-Value** |
| --- | --- | --- | --- | --- | --- | --- | --- | --- | --- | --- | --- |
| **Fixed effects** | evolutionary treatment | 3 | 3 | 66.0 | 6.57 | 0.0006 | **Random effects** | round | 20.73 | 3.61 | <0.0001 |
|  |  |  |  |  |  |  |  | round*treatment | -10.26 | 1.79 | <0.0001 |
|  |  |  |  |  |  |  |  | population[treatment] | -5.54 | 0.96 | <0.0001 |
|  |  |  |  |  |  |  |  | population*round[treatment] | -34.29 | 5.97 | <0.0001 |
|  |  |  |  |  |  |  |  | Residual | 174.19 | 30.32 |  |
|  |  |  |  |  |  |  |  | Total | 194.92 | 33.93 |  |

**Table S6.** Whole table summary for GLM of frequency of hosts harboring *B. subtilis*.

| **Model** | **-LogLikelihood** | **L-R ChiSquare** | **DF** | **Prob>ChiSq** |
| --- | --- | --- | --- | --- |
| Difference | 45.69 | 91.37 | 59 | 0.004 |
| Full | 357.30 |  |  |  |
| Reduced | 402.98 |  |  |  |

**Table S7.** Effect tests for GLM of frequency of hosts harboring *B. subtilis.*

| **Source** | **DF** | **L-R ChiSquare** | **Prob>ChiSq** |
| --- | --- | --- | --- |
| treatment | 2 | 9.45 | 0.009 |
| population[treatment] | 12 | 12.19 | 0.43 |
| round | 3 | 8.17 | 0.04 |
| treatment*round | 6 | 18.33 | 0.006 |
| population*round[treatment] | 36 | 50.72 | 0.05 |

**Table S8.** ANOVA table for host fecundity vs. *B. subtilis* CFU.

| **Source** | **DF** | **Sum of squares** | **Mean square** | **F Ratio** |
| --- | --- | --- | --- | --- |
| **Model** | 1 | 9.01 | 9.01 | 7.23 |
| **Error** | 14 | 17.45 | 1.25 | **Prob > F** |
| **C. Total** | 15 | 26.46 |  | 0.02 |

**Table S9.** *B. subtilis* spore formation in heat shocked and non-heat shocked nematodes.

| **Host** | **Assay environment** | **#spores** |
| --- | --- | --- |
| ancestor | heat shock | 0 |
| ancestor | heat shock | 0 |
| ancestor | heat shock | 0 |
| ancestor | no heat shock | 0 |
| ancestor | no heat shock | 4 |
| ancestor | no heat shock | 1 |
| B+H+ | heat shock | 0 |
| B+H+ | heat shock | 0 |
| B+H+ | heat shock | 0 |
| B+H+ | no heat shock | 0 |
| B+H+ | no heat shock | 21 |
| B+H+ | no heat shock | 0 |

**Figure S1. Fecundity means of evolved hosts after ten generations of selection**

The x-axis indicates the condition under which hosts evolved. To survey hosts after generation ten, nematodes from four populations of the indicated experimental evolution treatment were heat shocked at 34˚C on **a)** *B. subtilis* or **b)** *E. coli*. Each plate contained roughly 200 nematodes. The data is combined across two rounds. The dotted line and dashed line indicate the average value for the ancestral host on *B. subtilis* and *E. coli*, respectively. Error bars indicate the standard errors.

**Figure S2. Distribution of data points for fecundity means of evolved hosts in Figure 2**

Each data point is the average of three replicate plates for each of the five replicate populations from experimental evolution. The x-axis indicates the condition under which nematodes evolved. Nematodes from the four experimental treatments were heat shocked at 34˚C on **a)** *B. subtilis* **b)** or *E. coli*. Each plate contained roughly 200 nematodes. The data is combined across three rounds. **c)** Nematodes from the four experimental treatments were kept at 20˚C on *B. subtilis* (blue points) or *E. coli* (green points). Each shape represents a population from experimental evolution; the same shape across different treatments do not necessarily represent the same population. Each plate contained roughly 150 nematodes. The dotted line and dashed line indicate the average value for the ancestral host on *B. subtilis* and *E. coli*, respectively.

**Figure S3. Spread of *B. subtilis* by hosts into new environment**

After heat shock, 10 B+H+ hosts were transferred by platinum wire onto an unseeded NGM plate. Bacterial and nematode population growth was monitored for a week. (Top) Plates four days after transfer. (Bottom) Plates seven days after transfer. The blue circle on each plate indicates the initial spot where nematodes were placed. An asterisk indicates larvae were seen seven days after transfer. We observed qualitatively similar results when hosts were washed in buffer before being transferred to the unseeded NGMga plate (data not shown).

**Supplementary Methods**

Experimental evolution protocol

We began the experiment by surface sterilizing the ancestral host eggs using an established alkaline hypochlorite protocol (Stiernagle 2006) and putting roughly 700 larvae onto either *B. subtilis* (ten replicate populations) or *E. coli* (ten replicate populations). We kept them in an incubator at 20˚C. Once the nematodes reached adulthood (after three days), we heat shocked half of the *B. subtilis* plates and half of the *E. coli* plates at 34˚C for six hours, while the other half were left at 20˚C. The heat shocked plates were then left on the benchtop to cool down for 20 minutes, after which all nematodes were washed with M9 and transferred to OP50-GFP, where they were kept at 20˚C to recover from the heat shock and produce offspring. We used OP50-GFP as the recovery bacterium because it is phenotypically different from both *E. coli* OP50 and *B. subtilis* 168, but is still relatively neutral with respect to its effects on *C. elegans.* Furthermore, movement through the OP50-GFP lawn should remove any bacteria that were on the surface of nematodes when they were transferred over. Four days after heat shock, once the offspring had reproduced themselves, we transferred roughly 700 of their larvae onto fresh *B. subtilis* or OP50 *E. coli* revived from glycerol stock. We heat shocked them three days later, thus starting the next passage.

Bacterial colonization protocol

Briefly, we washed nematodes off their heat shocked plates with M9 into 1.5ml Eppendorf tubes. We then washed them three times with cold 0.01% Triton-X 100 in M9, then incubated them at 4˚C for 15 minutes. Afterwards we soaked them in 1:1000 diluted bleach for 15 minutes at 4˚C to further remove surface bacteria. We subsequently incubated them in ﻿0.25% sodium dodecyl sulfate (SDS) + 3% dithiothreitol (DTT) for 20 minutes, then transferred nematodes to a 96-well plate containing a ﻿small amount of sterile silicon carbide grit and 0.01% Triton X‐100 in M9. We then briefly disrupted the samples using a Qiagen TissueLyser II homogenizer and plated out the samples onto LB plates, quantifying the number of colony forming units (CFUs) two days later.

**References**

Stiernagle, T. 2006. Maintenance of *C. elegans*. WormBook 2006.
